# Vital Signs Prediction for COVID-19 Patients in ICU

**DOI:** 10.3390/s21238131

**Published:** 2021-12-05

**Authors:** Ahmed Youssef Ali Amer, Femke Wouters, Julie Vranken, Pauline Dreesen, Dianne de Korte-de Boer, Frank van Rosmalen, Bas C. T. van Bussel, Valérie Smit-Fun, Patrick Duflot, Julien Guiot, Iwan C. C. van der Horst, Dieter Mesotten, Pieter Vandervoort, Jean-Marie Aerts, Bart Vanrumste

**Affiliations:** 1 E-MEDIA, STADIUS, Department of Electrical Engineering (ESAT), Campus Group T, KU Leuven, 3000 Leuven, Belgium; Ahmed.youssefaliamer@kuleuven.be; 2 Measure, Model & Manage Bioresponses (M3-BIORES), Department of Biosystems, KU Leuven, 3000 Leuven, Belgium; jean-marie.aerts@kuleuven.be; 3 Limburg Clinical Research Center/Mobile Health Unit, Faculty of Medicine and Life Sciences, Hasselt University, 3500 Hasselt, Belgium; femke.wouters@uhasselt.be (F.W.); julie.vranken@uhasselt.be (J.V.); pauline.dreesen@uhasselt.be (P.D.); dieter.mesotten@zol.be (D.M.); pieter@vandervoort.mobi (P.V.); 4 Limburg Clinical Research Center/Mobile Health Unit, Ziekenhuis Oost-Limburg, 3600 Genk, Belgium; 5 Department of Anesthesiology, Ziekenhuis Oost-Limburg, 3600 Genk, Belgium; 6 Department of Cardiology and Future Health, Ziekenhuis Oost-Limburg, 3600 Genk, Belgium; 7 Department of Anesthesiology and Pain Management, Maastricht University Medical Centre+, 6229 HX Maastricht, The Netherlands; dianne.de.korte@mumc.nl (D.d.K.-d.B.); v.smit.fun@mumc.nl (V.S.-F.); 8 Department of Intensive Care, Maastricht University Medical Centre+, 6229 HX Maastricht, The Netherlands; frank.van.rosmalen@mumc.nl (F.v.R.); bas.van.bussel@mumc.nl (B.C.T.v.B.); iwan.vander.horst@mumc.nl (I.C.C.v.d.H.); 9 Service des Applications Informatiques, Centre Hospitalier Universitaire de Liège—CHU, 4000 Liège, Belgium; pduflot@chuliege.be; 10 Respiratory Medicine, Centre Hospitalier Universitaire de Liège—CHU, 4000 Liège, Belgium; j.guiot@chuliege.be

**Keywords:** COVID-19, ICU, vital signs prediction, kNN-LS-SVM

## Abstract

This study introduces machine learning predictive models to predict the future values of the monitored vital signs of COVID-19 ICU patients. The main vital sign predictors include heart rate, respiration rate, and oxygen saturation. We investigated the performances of the developed predictive models by considering different approaches. The first predictive model was developed by considering the following vital signs: heart rate, blood pressure (systolic, diastolic and mean arterial, pulse pressure), respiration rate, and oxygen saturation. Similar to the first approach, the second model was developed using the same vital signs, but it was trained and tested based on a leave-one-subject-out approach. The third predictive model was developed by considering three vital signs: heart rate (HR), respiration rate (RR), and oxygen saturation (SpO${}_{2}$). The fourth model was a leave-one-subject-out model for the three vital signs. Finally, the fifth predictive model was developed based on the same three vital signs, but with a five-minute observation rate, in contrast with the aforementioned four models, where the observation rate was hourly to bi-hourly. For the five models, the predicted measurements were those of the three upcoming observations (on average, three hours ahead). Based on the obtained results, we observed that by limiting the number of vital sign predictors (i.e., three vital signs), the prediction performance was still acceptable, with the average mean absolute percentage error (MAPE) being 12%,5%, and 21.4% for heart rate, oxygen saturation, and respiration rate, respectively. Moreover, increasing the observation rate could enhance the prediction performance to be, on average, 8%,4.8%, and 17.8% for heart rate, oxygen saturation, and respiration rate, respectively. It is envisioned that such models could be integrated with monitoring systems that could, using a limited number of vital signs, predict the health conditions of COVID-19 ICU patients in real-time.

## 1. Introduction

The recent COVID-19 pandemic shocked healthcare systems around the globe, highlighting the need for intelligent monitoring solutions. Intelligent monitoring solutions are needed to optimise the available resources in hospitals, more specifically in intensive care units (ICUs). During the first wave of the pandemic, ICUs experienced a shortage of beds, and healthcare workers were overloaded due to high admission rates and severely ill patients. Therefore, it became necessary to develop intelligent systems to optimise the available resources at hospitals, as well as the efforts of medical staff, to provide faster detection of patient deterioration. One key component of such an intelligent system involves data analytics of (big) medical data. Machine learning has become a popular and reliable analytical technique in recent years, especially in the medical domain. Many studies investigated hospitalised patients and ICU patients, for monitoring or mortality prediction [1,2,3,4,5,6,7,8]. Some of these studies investigated to what extent vital signs could inform on a patient’s clinical deterioration and adverse events [1,2]. Other studies focused on the mortality prediction of ICU patients and the relevant predictors [3,4,6]. For instance, in our previous study [4], we investigated the mortality prediction of ICU patients based on a set of vital signs, namely, heart rate, blood pressure, respiration rate, and oxygen saturation. The observation rates of these vital signs were hourly to bi-hourly measurements for each one. The main concept in that study was to engineer interpretable/simple features in combination with a linear hard margin support vector machine that can inform mortality prediction for ICU patients one to two days in advance. Moreover, in the study by Mahdavi et al. [6], they developed support vector machine models to predict the mortality risk of COVID-19 patients, based on demographic and laboratory variables/features obtained from a patient’s first day of admission. Another related approach involves vital sign predictors for hospitalised patients [7,8]. In our previous study [8], we developed predictive models to predict the future values of the same vital signs, up to three hours ahead on average. However, the population of that study comprised patients admitted to general wards (i.e., cardiology, post-surgical, and dialysis patients). Furthermore, in that study, the observations were sampled at 1 Hz using wearable devices (Somnotouch NIBP, http://www.somnomedics.eu (accessed on 28 November 2021)). Nevertheless, the acceptable predictions of both studies motivated us to integrate the two concepts, namely extracting simple features from low rate measurements of COVID-19 ICU patients to predict the near future values of the vital signs, up to three hours ahead on average.

In this study, we introduce, for the first time, machine learning predictive models to predict vital signs in COVID-19 patients. These machine learning models aim to predict a set of monitored vital signs, for patients in the ICU, in upcoming hours (i.e., three hours). Many machine learning algorithms are available that can be applied to such a case study. In their study, Han et al. [9] compared different machine learning algorithms (i.e., random forest, neural networks, and support vector machines) for a specific case study, and random forest outperformed. However, the machine learning algorithm we used in this study was the k-nearest neighbour least-squares support-vector machine (kNN-LS-SVM). This algorithm was chosen based on its efficient performance that was obtained in our previous study on vital signs predictors for hospitalised patients [8]. In addition, kNN-LS-SVM provides the advantage of localised learning algorithms, such as handling class imbalance, ambiguity, streaming analytics, and model personalisation [10]. The main focus of this study was to predict the future values of specific vital signs for upcoming observations (on average, three hours ahead) in COVID-19 ICU patients. These vital signs are mainly heart rate, respiration rate, and oxygen saturation. These vital signs were specified based on medical expert recommendations and constraints regarding the collected data. On the other hand, as portions of the collected data contain consistent measurements of blood pressure vital signs (i.e., systolic (SBP), diastolic (DBP), and mean arterial blood pressure (MAP)), we developed a predictive model considering these vital signs, as well for comparative reasons, to assess the influence of blood pressure (as a vital sign) on prediction performance.

## 2. Material and Methods

### 2.1. Data

Data used for developing and evaluating the models were collected for the WearIT4COVID Interreg project at the hospital Ziekenhuis Oost-Limburg (ZOL, Genk, Belgium), Maastricht University hospital MUMC+ (Maastricht, The Netherlands), and Le Centre hospitalier universitaire de Liège (CHU) (Liège, Belgium), during the first and second waves of the COVID-19 pandemic. The data were collected from the three hospitals with the details shown in Table 1. For ZOL, the data of COVID-19 positive patients admitted at the intensive care unit (ICU) were collected based on approval of the local ethics committee (20/003R). A total of 51 patients were included in this dataset with various lengths of stay at the ICU. The mean duration of ICU stay was 10.6 days (±8.87 days), and the mean of the entire hospital stay was 16.7 days (±9.27). For MUMC+, data collection in the Maastricht Intensive Care COVID (MaastrICCht) cohort was carried out by researchers from the ICU department. Ethical approval was obtained from the Medical Ethical Committee UM/azM (Maastricht). All patients diagnosed with COVID-19, and entering the ICU department, were included in the study; data were collected until patients were discharge from the ICU. Data from 50 patients were provided for analysis. The mean duration of ICU stay was 16.9 days (±16.3). For CHU Liège, the data of 84 patients were collected, and patients who were monitored for less than 24 h were excluded. The collected data from the three hospitals contained the vital parameters, demographic data (such as age and gender), comorbidities, and outcomes of 185 patients in total.

### 2.2. Methods

In this section, we address the machine learning algorithm that was used to develop the predictive model. The used machine learning algorithm was the hybrid localised learning algorithm k nearest neighbour least-squares support-vector machine (kNN-LS-SVM) for regression. This algorithm was introduced in our previous article [8] to predict the future values of the monitored vital signs of hospitalised patients in general wards using wearable sensors. The kNN-LS-SVM algorithm for regression was developed based on the studies of [10,11,12,13,14,15,16,17]. The integration of the kNN algorithm into the LS-SVM algorithm was proposed to build an efficient predictive model (LS-SVM) within a local region, which is determined by the kNN search algorithm. Localising the model is considered beneficial, to consider the local characteristics of the investigated region. In addition, such a localised learning approach could help to build updated models and provide online analytics [10].

#### Local Learning of SVMs

In this section, we begin by reviewing the main concepts behind support vector machines (SVMs) and localised learning approaches for SVMs.

SVMs are originally presented as binary classifiers that assign each datum instance $x\in R^{d}$ to one of two classes described by a class label y∈{−1,1}, based on the decision boundary that maximises the margin $2/{\left||w|\right|}_{2}$ between the two classes. Generally, a feature map $\phi :R^{d}\mapsto R^{p}$ is used to transform the geometric boundary between the two classes to a linear boundary $L:w^{T}\phi \left(x\right)+b=0$ in a feature space, for some weight vectors $w\in R^{p\times 1}$ and b∈R. The class of each instance can then be found by $y=\mathrm{sgn}\left(w^{⊤}\phi \left(x\right)+b\right)$, where sgn refers to the sign function.

Similar to the classification problems, regression models are obtained via estimating the boundary *L* based on a set of training examples $x_{i}$ (1≤i≤N) with corresponding output values $y_{i}\in R$. In particular, one is interested in parameters w and *b* that minimise a loss-function:

(1) $$\min_{w,\;b;\;\xi }\frac{1}{2}w^{⊤}w\;+C\sum_{i=1}^{N}\left(\xi_{i}+{\xi_{i}}^{*}\right),$$

and are subject to:

$$\begin{array}{c} y_{i}-w^{⊤}\phi \left(x_{i}\right)-b\leq \epsilon +\xi_{i},\;i=1,2,\ldots ,N\;,\\ w^{⊤}\phi \left(x_{i}\right)-y_{i}+b\leq \epsilon +{\xi_{i}}^{*},\;i=1,2,\ldots ,N,\\ \xi_{i},{\xi_{i}}^{*}\geq 0,\;\;\;i=1,2,\ldots ,N. \end{array}$$

The constant *C* in (1) denotes the *penalty term* that is used to penalise estimation error through the slack variables $\xi_{i}$ and ${\xi_{i}}^{*}$ outside Vapnik ϵ-sensitivity loss function in the optimisation process.

LS-SVM’s are obtained by using a least-squares error loss function [16]:

(2) $$\min_{w,\;b;\;e}\frac{1}{2}w^{⊤}w\;+\frac{1}{2}\gamma \sum_{i=1}^{N}{e_{i}}^{2},$$

such that

$$\begin{array}{c} y_{i}=w^{⊤}\phi \left(x_{i}\right)+b+e_{i},\;i=1,2,\ldots ,N. \end{array}$$

where γ is the regularisation constant for LS-SVM. The optimisation procedure introduces errors $e_{i}$, such that $1-e_{i}$ is proportional to the signed distance of $x_{i}$ from the decision boundary. In fact, the non-negative slack variable constraint is removed and the solution of the optimisation problem can be obtained by a set of linear equations, reducing computational efforts [16].

Local learning approaches build models that fit the data in the local neighbourhood around a test example and by locally adjusting the model parameters to the properties of the data [14].

While global SVMs consider the same weights for all training instances in the optimisation process (2), local learning approaches allow for the training samples near a test point to be more influential than others. Localised learning approaches of SVMs [10] are based on weighting functions $\lambda (x_{s},x_{i})$ that express the similarity between the feature vectors of the *i*-th data point $x_{i}$ and a test instance $x_{s}$. For an LS-SVM, this leads to the following cost function:

(3) $$\min_{w,\;b;\;e}\frac{1}{2}w^{⊤}w+\frac{1}{2}\gamma \sum_{i=1}^{N}\lambda \left(x_{s},x_{i}\right)e_{i}^{2},$$

such that

$$\begin{array}{c} y_{i}=w^{⊤}\phi \left(x_{i}\right)+b+e_{i},\;i=1,2,\ldots ,N. \end{array}$$

where $y_{i}$ is a real number. Weighted least-squares support vector machines [17] use a similar approach, but here, a different weighting function can be used for any given test point $x_{s}$. In this work, we study a binary valued similarity criterion:

$$\lambda \left(x_{s},x_{i}\right)=\left\{\begin{array}{cc} 1 & if\;||x_{s}-x_{i}{\left|\right|}_{2}\leq r_{s}\\ 0 & otherwise, \end{array}\right.$$

where $r_{s}$ is the *K*-th smallest distance among $\left\{||x_{s}-x_{i}||;1\leq i\leq N\right\}$. This formulation leads to the hybrid KNN-LS-SVM method that we applied on the time-series prediction approach. In particular, a regression model was built for each test example using only the training examples located in the vicinity of the test example [11].

KNN-LS-SVM has the additional advantage of sparseness. Indeed, for an LS-SVM or the localised version that uses a continuous similarity function, all input data are required to construct the separating hyperplane [17]. This can be seen by solving the optimisation problem (3). Using the method of the Lagrangian multipliers, we find:

$$\begin{array}{c} L\left(w,b,e;\alpha \right)=\frac{1}{2}{∥w∥}_{2}^{2}+\frac{1}{2}\gamma \sum_{i=1}^{N}\lambda \left(x_{s},x_{i}\right)e_{i}^{2}-\\ \sum_{i=1}^{N}\alpha_{i}\left(w^{⊤}\phi \left(x_{i}\right)+b+e_{i}-y_{i}\right), \end{array}$$

where $\alpha_{i}$ are the *Lagrangian* multipliers. Thus, for a KNN-LS-SVM, the sparseness characteristic is returned to the LS-SVM, as only the neighbour data points contribute to the model. In an online learning mode, this sparseness will result in a computational advantage compared to LS-SVM.

**Figure 1:**
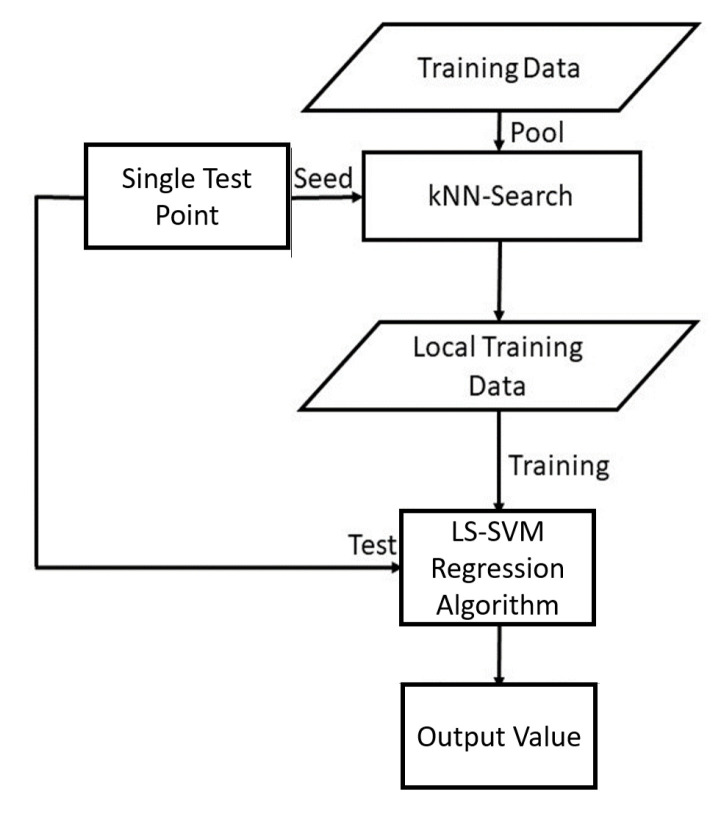
A flow chart illustrating the localised learning algorithm of KNN-LS-SVM for regression (adapted from ref. [8]).

The illustration of the kNN-LS-SVM regression algorithm is shown in Figure 1. The algorithm of KNN-LS-SVM is implemented as follows:

1. Given a test example $x_{s}$, compute distances to all training examples and pick the nearest *K* neighbours.
2. Train the LS-SVM model with the *K* nearest neighbours.
3. Use the resulting regressor to estimate the output of $x_{s}$.

The parameter *K* and the distance metric (e.g., Euclidean, Mahalanobis, or Chebyshev) are additional hyperparameters next to the kernel width $\sigma_{0}$ and the penalty term γ that are optimised in a cross-validation approach [10]. One challenge faced in finding the nearest neighbour in a continuously increasing data pool is the search complexity. However, several advanced search algorithms were developed to reduce this complexity [18].

## 3. Results

In this section, we introduce the prediction results of the developed predictive models following two test approaches. The first approach is the 80/20 approach, in which 80% (7100 datapoints) of the shuffled data are used to train the model, and the remaining 20% (1845 datapoints) of the data are used to test the model. The other approach is leave-one-subject-out, in which for each subject, this subject’s data (on average 50 datapoints) is excluded from the training set of the model to be used as the test set.

The developed models in this study are based on data from the three hospitals. Moreover, the results from the three hospitals together are consistent with the results from each hospital individually. Therefore, in this section, we focus on the results from the merged data of the three hospitals together. Prior to applying the localised learning approach of kNN-LS-SVM, a set of statistical features were extracted from the raw measurements of each vital sign. The extracted features were the minimum, maximum, mean, median, standard deviation, and energy. These features were extracted from a time window of three observations to predict the upcoming vital sign values up to three observations ahead (approximately three hours on average, as the observation rate is not uniform). After extracting the aforementioned features, we developed five predictive models. The predictive models were evaluated based on the mean absolute percentage error (MAPE) which is calculated by the formula MAPE = $\frac{1}{n}\sum_{t=1}^{n}∥\left(A_{t}-P_{t}\right)/A_{t}∥$, where *n* is the number of observations, $A_{t}$ and $P_{t}$ are the actual and predicted values at time *t*.

**Figure 2:**
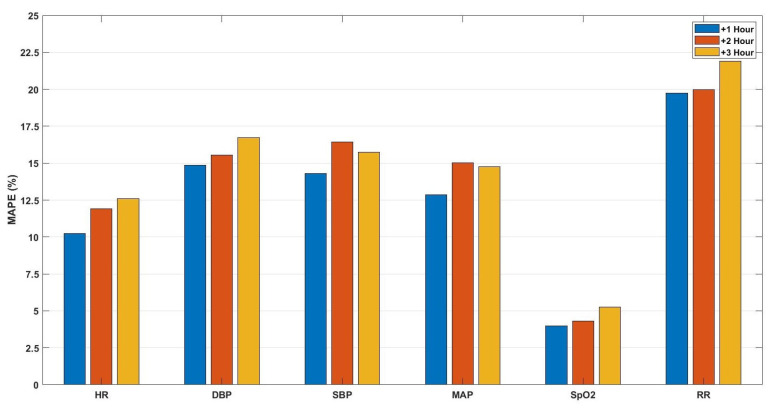
The mean absolute percentage error of the predictions for the upcoming three hours (+1, +2, +3 h) of the vital signs: heart rate (HR), diastolic (DBP), systolic (SBP), mean arterial blood pressure (MAP), oxygen saturation (SpO${}_{2}$), and respiration rate (RR), respectively, based on the hourly to bi-hourly observation rate.

For the first predictive model, we aimed to predict the future values of the vital signs for the upcoming three observations (on average three hours ahead) based on hourly to bi-hourly observation rate. These vital signs were heart rate, respiration rate, oxygen saturation, and blood pressure (systolic, diastolic, and mean arterial). For this predictive model, the features were extracted from all of these vital signs and forming vectors, with (6 features × 6 vital signs) 36 dimensions. In other words, all vital signs contributed to predicting each vital sign. The prediction performance is shown in Figure 2 in terms of mean absolute percentage error (MAPE).

**Figure 3:**
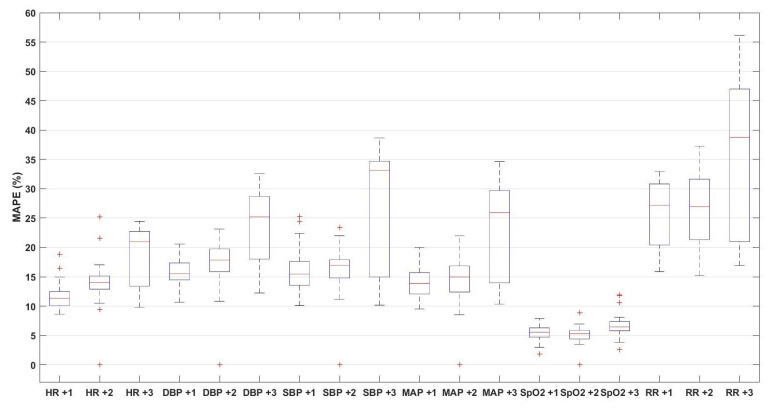
The box-plot of leave-one-subject-out predictions of the vital signs of heart rate (HR), diastolic (DBP), systolic (SBP), mean arterial blood pressure (MAP), oxygen saturation (SpO${}_{2}$), and respiration rate (RR) for the upcoming three observations (on average 3 h ahead).

The second predictive model is similar to the first, however, it is trained/tested based on the leave-one-subject-out approach. As shown in Figure 3, the box-plots of the resulting MAPE for the predictions of the six vital signs are depicted.

**Figure 4:**
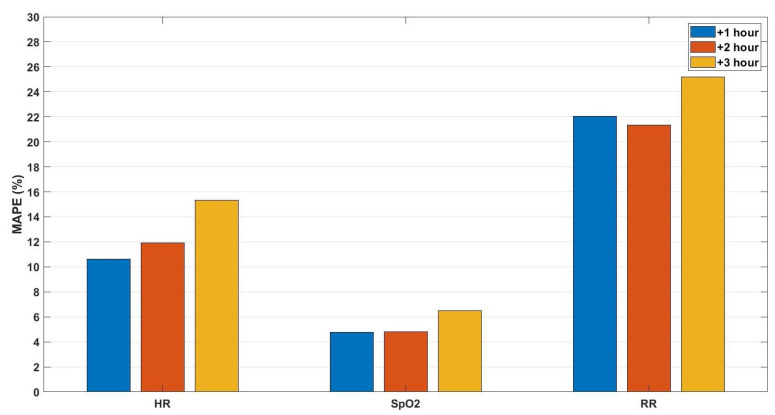
The mean absolute percentage error of the predictions for the upcoming three hours (+1, +2, +3 h) of the vital signs: heart rate (HR), oxygen saturation (SpO${}_{2}$), and respiration rate (RR), respectively, based on hourly to bi-hourly observation rate.

**Figure 5:**
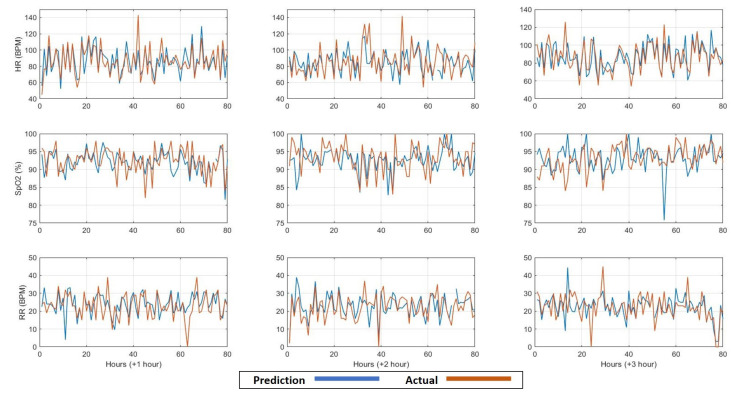
The comparison between the predicted and the actual values for the upcoming three hours (+1, +2, +3 h) of the vital signs: heart rate (HR), oxygen saturation (SpO${}_{2}$) and respiration rate (RR), respectively, based on hourly to bi-hourly observation rate.

For the third predictive model, we aimed to predict the future values of only three vital signs for the upcoming three observations (three hours on average). These vital signs were heart rate, respiration rate, oxygen saturation. These three vital signs were selected as they were recorded in a more consistent way compared to blood pressure. Furthermore, these vital signs could be easily measured using wearable sensors in contrast with blood pressure. For this predictive model, the features were extracted from the three vital signs as well forming vectors of (6 features × 3 vital signs) 18 dimensions. The prediction performance is shown in Figure 4 in terms of MAPE. Moreover, examples of the comparison between the predicted and the actual values of the three vital signs for the upcoming three hours are shown in Figure 5.

**Figure 6:**
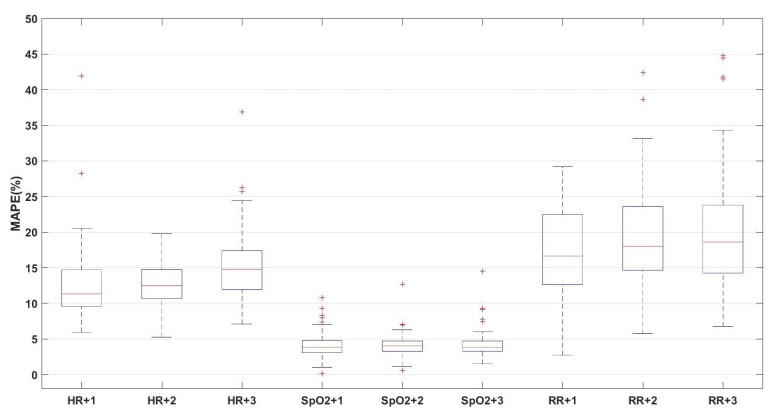
The box-plot of the leave-one-subject-out prediction of the vital signs of heart rate (HR), oxygen saturation (SpO${}_{2}$), and respiration rate (RR) for the upcoming three observations (on average 3 h ahead).

Similar to the third model, for the fourth predictive model, it was based on the three vital signs (heart rate, respiration rate, and oxygen saturation), but it was trained and tested using the leave-one–subject-out approach. The box-plot of the resulting MAPE of the predictions are shown in Figure 6.

**Figure 7:**
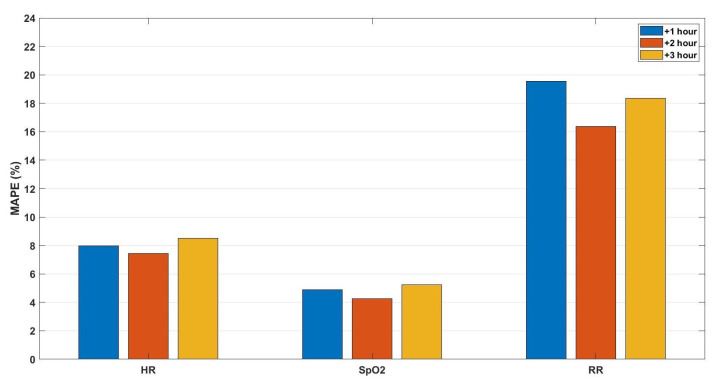
The mean absolute percentage error of the predictions for the upcoming three hours (+1, +2, +3 h) of the vital signs: heart rate (HR), oxygen saturation (SpO${}_{2}$), and respiration rate (RR), respectively, based on an observation rate of one observation/5 min.

**Figure 8:**
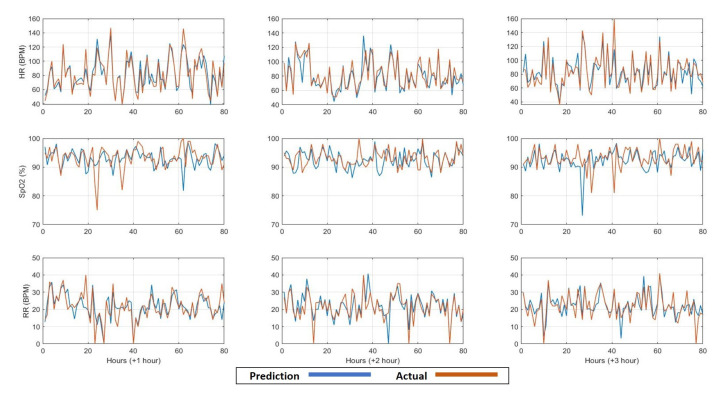
The comparison between the predicted and the actual values for the upcoming three hours (+1, +2, +3 h) of the vital signs: heart rate (HR), oxygen saturation (SpO${}_{2}$), and respiration rate (RR), respectively, based on observation rate of one observation/5 min.

Finally, the fifth predictive model predicted the future values of heart rate, oxygen saturation, and respiration rate for the upcoming three observations (on average three hours). However, the observation rate, in this case, was one observation/5 min. Therefore, the same features were extracted from the same time windows, but with a larger number of observations (i.e., 12 observations per hour), forming vectors with (3 h × 6 features × 3 vital signs) 54 dimensions to predict the 1-hour average values for the upcoming three hours. The prediction performance is shown in Figure 7 in terms of MAPE. Furthermore, in Figure 8, we show some examples of the comparison between the predicted and the actual values of the three vital signs with observation rate, one observation/5 min, for the upcoming three hours.

**Figure 9:**
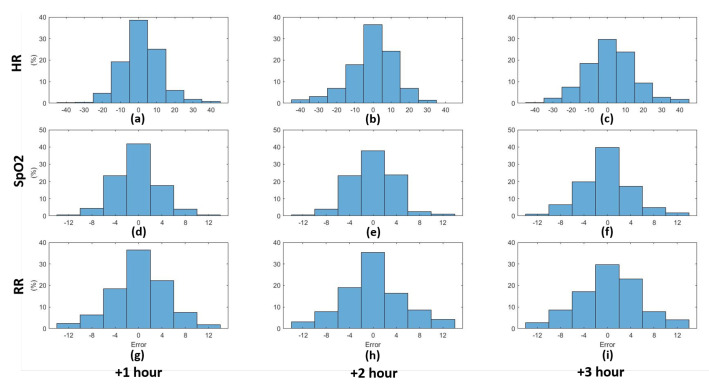
Normalised histograms of the error of the predicted values for the three vital signs for the upcoming three hours based on hourly to bi-hourly observations. Each row represents one of the three vital signs: heart rate, oxygen saturation, and respiration rate respectively. Each column represents the prediction hour: +1, +2, +3 h, respectively. (**a**) HR +1 h, (**b**) HR +2 h, (**c**) HR +3 h, (**d**) Sp$O_{2}$ +1 h, (**e**) Sp$O_{2}$ +2 h, (**f**) Sp$O_{2}$ +3 h, (**g**) RR +1 h, (**h**) RR +2 h, (**i**) RR +3 h.

**Figure 10:**
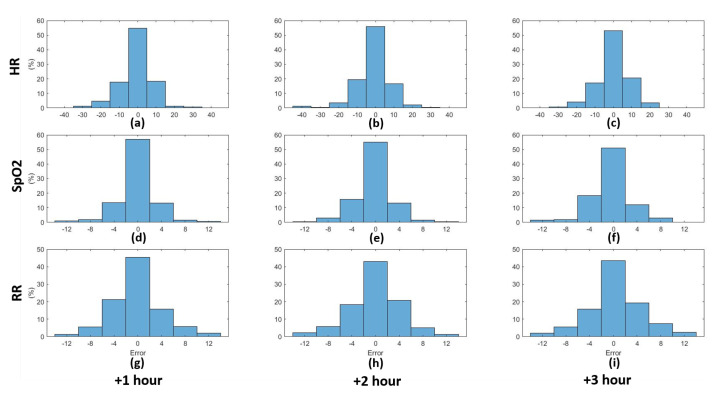
Normalised histograms of the error of the predicted values for the three vital signs for the upcoming three hour based observation rate of one observation/5 min. Each row represents one of the three vital signs: heart rate, oxygen saturation, and respiration rate respectively. Each column represents the prediction hour: +1, +2, +3 h respectively. (**a**) HR +1 h, (**b**) HR +2 h, (**c**) HR +3 h, (**d**) Sp$O_{2}$ +1 h, (**e**) Sp$O_{2}$ +2 h, (**f**) Sp$O_{2}$ +3 h, (**g**) RR +1 h, (**h**) RR +2 h, (**i**) RR +3 h.

**Figure 11:**
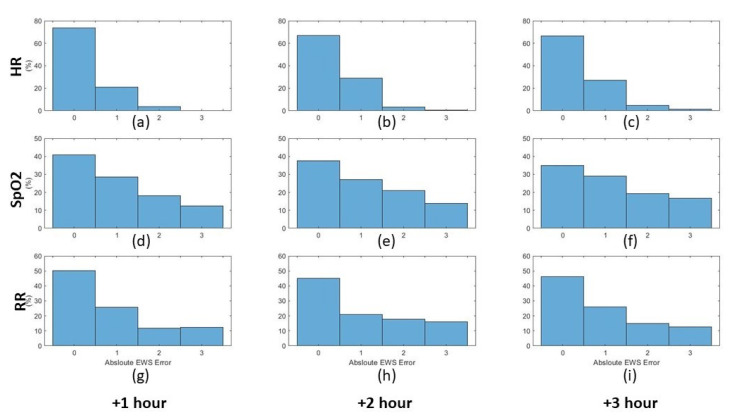
Normalised histograms of the absolute EWS error of the predicted values for the three vital signs for the upcoming three hours based on hourly to bi-hourly observations. Each row represents one of the three vital signs: heart rate, oxygen saturation, and respiration rate, respectively. Each column represents the prediction hour: +1, +2, +3 h, respectively. (**a**) HR +1 h, (**b**) HR +2 h, (**c**) HR +3 h, (**d**) Sp$O_{2}$ +1 h, (**e**) Sp$O_{2}$ +2 h, (**f**) Sp$O_{2}$ +3 h, (**g**) RR +1 h, (**h**) RR +2 h, (**i**) RR +3 h.

**Figure 12:**
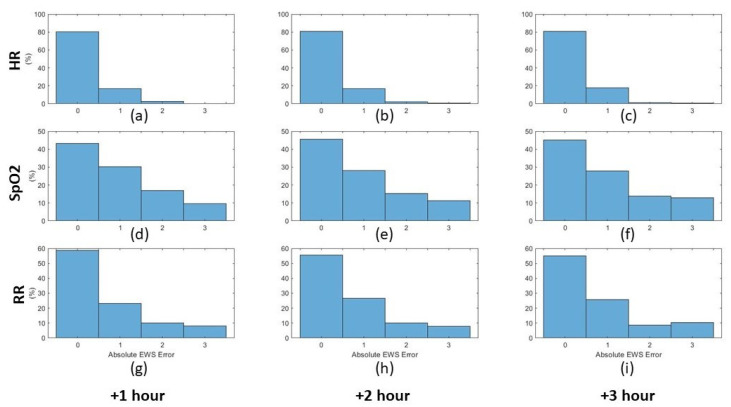
Normalised histograms of the absolute EWS error of the predicted values for the three vital signs for the upcoming three hours based on the observation rate of one observation/5 min. Each row represents one of the three vital signs: heart rate, oxygen saturation, and respiration rate, respectively. Each column represents the prediction hour: +1, +2, +3 h, respectively. (**a**) HR +1 h, (**b**) HR +2 h, (**c**) HR +3 h, (**d**) Sp$O_{2}$ +1 h, (**e**) Sp$O_{2}$ +2 h, (**f**) Sp$O_{2}$ +3 h, (**g**) RR +1 h, (**h**) RR +2 h, (**i**) RR +3 h.

Based on the obtained predictions, the histograms of the obtained error between the predicted and actual values were calculated for both the third and fifth predictive models (cf. supra) as depicted in Figure 9 and Figure 10 for the three vital signs (heart rate, oxygen saturation, and respiration rate) for the upcoming three hours. After performing the prediction, the early warning score components for the predicted vital signs were calculated for the predicted and the actual values. The absolute error between the predicted and the actual EWS components was calculated for the third and fourth predictive models based on the ranges depicted in Table 2. The normalised histograms of the absolute EWS error are shown in Figure 11 and Figure 12 for the third and fifth predictive models, respectively.

## 4. Discussion

Based on the obtained results, we can compare the performance of the different models. For example, by comparing the first and second predictive models, it is noticeable that the performance of the first model, in terms of MAPE, is better for all vital signs than the median MAPE values of the second model, which indicates that the performance is better when the same-person data are considered in the training set.

By comparing the prediction performance of the first and third models, as both are based on hourly to bi-hourly observation rates, we notice that the HR and SpO${}_{2}$ prediction error for the first model is similar to that of the third model for the first and second-hour predictions, without a statistical significance (at 5% significance level), and less for the third hour without significance. This similarity can indicate that, for these models, the considered blood pressure vital signs are unnecessary for both HR and SpO${}_{2}$. However, for RR, the prediction performance of the first model (MAPE = 19.8%, 20%, and 22%) is better than that of the third model (MAPE = 22%, 21.3%, 25.2%) with significance at 10% significance level. This outperformance indicates that blood pressure vital signs can be informative to predict the respiration rate. Given these results, we can conclude that blood pressure vital signs did not significantly influence the prediction performance for both heart rate and oxygen saturation in this setup. On the other hand, respiration rate prediction is influenced by the absence of blood pressure parameters. However, we should notice that the respiration rate for the considered population is biased because at some moment(s) during their stays, patients were coupled to mechanical ventilation, which makes the prediction of respiration rate rather ambiguous. Moreover, it is worth mentioning that for all predictive models, the MAPE values of RR are with the highest values, as the range of RR is small compared to other vital signs (15 ± 6 BPM). Hence, an error with 1 BPM represents approximately 5% of the original value.

For the third and fourth models, we observed that the performance of both models is approximately similar without statistical significance by comparing the average MAPE values in Figure 4 and the average values of the box plots in Figure 5. This result indicates that the models based on the three vital signs are not notably influenced by the same-person data.

By comparing the performance of the third and fifth models, we observe a significant enhancement in the prediction performance of the fifth model compared to the third model, as shown in Figure 4 and Figure 6, which indicates that by increasing the observation rate, the prediction performance will enhance.

A general note for SpO${}_{2}$ prediction is that the variation range of SpO${}_{2}$ is between 85 to 100% for this population. Therefore, an absolute percentage error of 5% represents almost one-third of the variation range, which can be considered as a limitation of the prediction performance.

Furthermore, the normalised histograms of the prediction errors as shown in Figure 9 and Figure 10 show the outperformance of the fifth model compared to the third model. More specifically, for HR, the error frequency within the lowest error range (−5 to 5 BPM) is approximately 20% higher for the fifth model than for the third model, which indicates a lower error frequency for the higher error values. Similarly, for both SpO${}_{2}$ and RR, the error frequency within the lowest error range (−2 to 2%) and (−2 to 2 BPM) respectively, is higher for the fifth model than the third model with approximately 10%. Furthermore, by applying the F-test to test the significance between the different distribution variances, it is observed that the significance (5% significance level) is observed between each distribution in Figure 9 and its equivalent in Figure 10. This significance approves the improvement in the error distributions that can be obtained by increasing the observation rate. Moreover, by comparing the absolute EWS error between the two models, it is noticeable that the prediction error for the third model is lower than that of the second model for the three parameters for the three prediction horizons, as shown in Figure 11 and Figure 12.

## 5. Conclusions

Conclusively, in this study, we introduced for the first time machine learning predictive models to predict the future values of COVID-19 patients’ vital signs at ICU. These predictive models have shown promising performances with a few parameters (i.e., three vital signs) in predicting their values for the upcoming three hours. This performance is evaluated in terms of MAPE and it is on average 12%,5% and 21.4% for HR, SpO${}_{2}$, and R,R respectively. Moreover, the performances of the predictive models based on three vital signs show robustness in training the model, whether considering the same-person data or not. Ultimately, increasing the observation rate could enhance the prediction performance to be on average 8%,4.8%, and 17.8% for heart rate, oxygen saturation, and respiration rate, respectively. Ultimately, the obtained results show promising performance for the particular profile of COVID-19 ICU patients.

For future work, having a high data rate (e.g., 1 Hz) may enhance the performance. Moreover, we would suggest to test our approach on COVID-19 patients outside ICU for real-time monitoring. Such real-time monitoring and prediction can lighten the workload on the medical staff in critical times during the pandemic. Furthermore, for larger datasets, dividing the group of patients into sub-groups based on their comorbidities may enhance the prediction performance. Ultimately, one of the main hurdles that may make it challenging to provide real-time analytics is the limited data storage resources at most hospitals. Hence, upgrading data storage facilities at hospitals would be a game-changer for intelligent medical systems. 

## Figures and Tables

**Table 1:** The demographic presentation and most frequent comorbidities (prior to or acquired during their COVID infection) of the patients admitted to ICU in the three hospitals: ZOL, MUMC+, and CHU de Liège.

	ZOL (51 Patients)	MUMC+ (50 Patients)	CHU de Liège (84 Patients)
Demographic and comorbidity parameters	Descriptive statistics		
**Age (mean ± std)**	65.84 ± 12.44	67.02 ± 1.28	69 ± 12.16
**Gender (male, %)**	62.7%	80.0%	68%
**Height (cm; mean ± std)**	166.63 ± 12.11	176.3 ± 8.3	168 ± 26.53
**Weight (kg; mean ± std)**	83.76 ± 16.34	85.91 ± 13.68	81.45 ± 20.01
**Smoking status (%)**	Never: 66% Smoker: 6.4% Former smoker: 27.7%	Never: 90% Smoker: 6.0% Former smoker: 4.0%	Never: 50% Smoker: 6% Former Smoker: 12%
**Cardiovascular disease (%)**	17.6%	4.0%	23% (yes) 22% (no) 55% (unknown)
**Diabetes (%)**	27.5%	18.0%	51% (yes) 21% (no) 28% (unknown)
**Arterial hypertension (%)**	43.1%	16.0%	61% (yes) 14% (no) 25% (unknown)
**Cerebrovascular accident or transient ischaemic attack (%)**	5.9%	2.0%	(unknown)
**Kidney insufficiency (%)**	60.8%	0.0%	(unknown)
**Heart Failure (%)**	29.4%	4.0%	(unknown)
**NYHA classification**	II: 8.3% III: 8.3%	6.0%	(unknown)
**Myocardial infarction (%)**	9.8%	42%	(unknown)
**PCI/PTCA**	9.8%	67.02 ± 1.28	(unknown)
**COPD**	11.8 %	80.0 %	24% (yes) 29% (no) 47% (unknown)
**Asthma (%)**	7.8%	176.3 ± 8.3	9% (yes) 38% (no) 53% (unknown)
**Intubated**	23 (46%)	50 (100%)	38 (46%)
**O${}_{2}$ Mask/Nasal Cannula**	40 (78%)	(Unknown)	71 (86%)

**Table 2:** Early warning score system based on Ziekenhuis Oost-Limburg (ZOL) Hospital.

SCORE	3	2	1	0	1	2	3
Temperature (${}^{\circ }$C)		<35.1	35.1–36.5	36.6–37.5	>37.5		
Heart Rate (BPM)		<40	40–50	51–100	101–110	111–130	>130
Respiration Rate (BPM)		<9		9–14	15–20	21–30	>30
Oxygen Saturation (%)	<91	91–93	94–95	>95			
Systolic Blood Pressure (mmHg)	<70	70–80	81–100	101–180	180–200	>200	

## Data Availability

Dataset supporting the study may be obtained upon request to Liege University Hospital, Maastricht UMC+, and Ziekenhuis Oost-Limburg in accordance with applicable laws.
